# Complete Genome Sequence of an Efficient Rhizobium leguminosarum bv. viciae Strain, A1

**DOI:** 10.1128/MRA.00249-20

**Published:** 2020-05-07

**Authors:** Alexey M. Afonin, Emma S. Gribchenko, Anton S. Sulima, Vladimir A. Zhukov

**Affiliations:** a Laboratory of Genetics of Plant-Microbe Interactions, All-Russia Research Institute for Agricultural Microbiology, St. Petersburg, Russia; Indiana University, Bloomington

## Abstract

Rhizobium leguminosarum strain A1 is used in inoculation experiments with a wide range of pea (Pisum sativum L.) lines. In this study, we report the genome sequence of strain A1, consisting of a 5.06-Mbp circular chromosome and circular plasmids ranging from 804,800 bp to 154,738 bp long.

## ANNOUNCEMENT

Strain A1 was first isolated in 1986 from the nodules of a pea plant cultivated in a field adjacent to the All-Russia Research Institute for Agricultural Microbiology (ARRIAM) (1). It was shown to possess useful qualities, such as being able to nodulate a wide range of pea varieties, including those carrying the *Sym2A* allele inherent in line NGB2150 (JI1357, WBH 2150) with the Afghan or Afghanistan phenotype, and it was capable of overcoming competitive nodulation blocking, making it an efficient strain for agricultural use (1, 2). Additionally, it was shown to produce large quantities of various lipochitin oligosaccharides (LCOs), including Nod factors (3). The strain was observed to lose symbiotic properties much faster than other strains used in inoculation experiments (e.g., RCAM1026) (data not shown). The genetic factors responsible for the multiple unique features remained undiscovered.

Plants of Pisum sativum line NGB2150 were inoculated with the A1 strain, pink nodules were harvested, bacteria were isolated as described previously (4), and the strain was preserved in 10% glycerol at −80°C. For DNA isolation, the strain was revived on solid tryptone-yeast extract (TY) medium. One colony was chosen for subsequent procedures. The strain was cultivated in 50 ml of liquid TY medium in a 100-ml flask at 28°C and 200 rpm (5). The culture was harvested after 48 h of incubation. DNA was isolated using the phenol-chloroform method (6) and quantified with a spectrophotometer (BioSpec-mini; Shimadzu, Japan). The required library absorption parameters were an *A*_260_/*A*_280_ ratio of ∼2 and an *A*_260_/*A*_230_ ratio of >1.8.

Long-read whole-genome sequencing was performed using a MinION sequencer (Oxford Nanopore, United Kingdom) in the ARRIAM. The SQK-LSK109 ligation sequencing kit and the EXP-NBD104 native barcoding expansion 1-12 kit were used to prepare the library according to the manufacturer’s instructions, omitting the DNA-shearing step. The reads were base called and demultiplexed using Guppy base caller (v. 3.3.0). The resulting read *N*_50_ value was 31,627 bp, with a total read length of 0.2 Gbp and estimated coverage of 25×. The Flye pipeline (v. 2.6) (7) was used to assemble the Nanopore reads. The resulting assembly was corrected four times using Racon (v. 1.3.2) (8) (with the modifiers -m 8 -x -6 -g -8 -w 500), followed by a single polish using the medaka program (v. 0.10.0) with default parameters.

Short-read whole-genome sequencing of the strain was carried out on an Illumina system with the TruSeq DNA PCR-free kit; in total, 8,721,349 bp of 2 × 150-bp sequence reads were generated. The reads were quality trimmed and adapter sequences and possible contaminants were removed as described previously (9); after filtering, the expected coverage was about 218×. The short reads were used to polish the assembled genome using the Pilon (v. 1.22) algorithm (10). PGAP was used to annotate the assembled transcripts (11).

The genome of strain A1 consists of 6 replicons, including 1 chromosome and 5 plasmids. The statistics for the amplicons are presented in Table 1.

**TABLE 1:** Characteristics of the replicons of strain Rhizobium leguminosarum A1

Amplicon	Length (bp)	No. of CDSs	No. of tRNAs	GC content (%)	Accession no.
A1 chromosome	5,067,161	4,943	52	60.94	CP049730
pRL12	804,800	745	0	60.71	CP049731
pRL11	661,136	656	2	60.70	CP049732
pRL10	632,304	588	0	60.87	CP049733
pRLa11	328,507	321	0	57.67	CP049734
pRLa12	154,738	185	0	56.48	CP049735

The circularity of all of the assembled fragments was reported by the Flye assembly pipeline and verified by mapping the long reads to the assembled fragments using Minimap2 (12), with the map-ont mapping mode, and inspecting the coverage uniformity. The chromosome was rotated so that the *dnaA* gene was placed at the start of the sequence; for each plasmid, a *repABC* operon was located and placed at the start of the sequence.

The relation to other Rhizobium leguminosarum strains was determined using the average nucleotide identity (ANI) method (13). The strain closest to the A1 strain was strain RCAM1026 (97.2%), which is used for inoculation studies in the ARRIAM (14), placing the strain in genospecies C, according to reference 15.

The coding sequences (CDSs) predicted by the PGAP were annotated using eggNOG mapper (v. 2) (16) with the eggNOG (v. 5.0) database (17). Additionally, the CDSs were compared to the latest version of the UniProt Swiss-Prot curated database (18). BLASTp (v. 2.9.0+) was used to search the database with the value 1e−10, and the identity threshold was set at 60% (19).

The plasmid pRLa11 contains 13 predicted Nod factor-associated genes (*nodA* [NCBI accession number WP_017958626.1], *nodJ* [WP_017958630.1], *nodN* [WP_138333862.1], *nodM* [WP_138333863.1], *nodL* [WP_138333865.1], *nodE* [WP_138333867.1], *nodF* [WP_138333869.1], *nodD1* [WP_138333871.1], *nodB* [WP_138333873.1], *nodC* [WP_138333874.1], *nodI* [WP_138333928.1], *nodT* [WP_165586599.1], and *nodX* [WP_138333876.1]), as described previously (3). Additionally, 5 nodulation genes were found on the chromosome (*nodT* [WP_018068951.1], *nodG* [WP_018070560.1], *nodT* [WP_130672970.1], *nodN* [WP_130673101.1], and *nodL* [WP_130673413.1]), 1 on pRL10 (*nodT* [WP_018071823.1]), and 1 on pRLa12 (*tolC* family protein [WP_165586630.1]). The gene present in four distinct copies was *nodT*, which was previously reported to be involved in the secretion of small molecules and, presumably, nodulation factors (20). Multiple clusters of genes annotated as *vir* genes belonging to type IV secretion systems were found on the pRLa11 and pRLa12 plasmids.

The large number of *nod* genes found in the genome is probably responsible for the previously observed high variability of the LCOs produced (3). Multiple genes encoding secretion systems, including the four copies of the *nodT* gene, may be the cause of the increased Nod factor excretion by the strain described previously (3). The full-genome sequence of this strain will be useful for further investigation of the symbiotic properties of this strain.

### Data availability.

The assemblies and sequence data have been deposited in the NCBI database. The BioProject number is PRJNA609819, the BioSample number is SAMN14260269, and the assembly accession numbers are CP049730 to CP049735. The raw Illumina data can be found under number SRR11216745, and the demultiplexed fastq file, with barcodes removed, from the MinION runs can be found under number SRR11216744. This announcement describes the first version of the genome assembly.
